# Interactive effects of CDKN2B‐AS1 gene polymorphism and habitual risk factors on oral cancer

**DOI:** 10.1111/jcmm.17966

**Published:** 2023-09-19

**Authors:** Jung‐Chun Yeh, Yi‐Tzu Chen, Ying‐Erh Chou, Shih‐Chi Su, Lun‐Ching Chang, Yen‐Lin Chen, Chiao‐Wen Lin, Shun‐Fa Yang

**Affiliations:** ^1^ School of Dentistry, Chung Shan Medical University Taichung Taiwan; ^2^ Department of Dentistry Chung Shan Medical University Hospital Taichung Taiwan; ^3^ School of Medicine, Chung Shan Medical University Taichung Taiwan; ^4^ Department of Medical Research Chung Shan Medical University Hospital Taichung Taiwan; ^5^ Whole‐Genome Research Core Laboratory of Human Diseases, Chang Gung Memorial Hospital Keelung Taiwan; ^6^ Department of Dermatology, Drug Hypersensitivity Clinical and Research Center Chang Gung Memorial Hospital Linkou Taiwan; ^7^ Department of Mathematical Sciences Florida Atlantic University Boca Raton Florida USA; ^8^ Institute of Oral Sciences, Chung Shan Medical University Taichung Taiwan; ^9^ Institute of Medicine, Chung Shan Medical University Taichung Taiwan

**Keywords:** cyclin dependent kinase inhibitor 2B antisense RNA 1, long noncoding RNA, oral squamous cell carcinoma, single‐nucleotide polymorphism

## Abstract

Oral squamous cell carcinoma (OSCC) is a common malignant disease associated with a high mortality rate and heterogeneous disease aetiology. Cyclin dependent kinase inhibitor 2B antisense RNA 1 (CDKN2B‐AS1), is a long noncoding RNA that has been shown to act as a scaffold, sponge, or signal hub to promote carcinogenesis. Here, we attempted to assess the effect of *CDKN2B‐AS1* single‐nucleotide polymorphisms (SNPs) on the susceptibility to OSCC. Five *CDKN2B‐AS1* SNPs, including rs564398, rs1333048, rs1537373, rs2151280 and rs8181047, were analysed in 1060 OSCC cases and 1183 cancer‐free controls. No significant association of these five SNPs with the risk of developing OSCC was detected between the case and control group. However, while examining the clinical characteristics, patients bearing at least one minor allele of rs1333048 (CA and CC) were more inclined to develop late‐stage (stage III/IV, adjusted OR, 1.480; 95% CI, 1.129–1.940; *p* = 0.005) and large‐size (greater than 2 cm in the greatest dimension, adjusted OR, 1.347; 95% CI, 1.028–1.765; *p* = 0.031) tumours, as compared with those homologous for the major allele (AA). Further stratification analyses demonstrated that this genetic correlation with the advanced stage of disease was observed only in habitual betel quid chewers (adjusted OR, 1.480; 95% CI, 1.076–2.035; *p* = 0.016) or cigarette smokers (adjusted OR, 1.531; 95% CI, 1.136–2.063; *p* = 0.005) but not in patients who were not exposed to these major habitual risks. These data reveal an interactive effect of *CDKN2B‐AS1* rs1333048 with habitual exposure to behavioural risks on the progression of oral cancer.

## 1 INTRODUCTION

Oral tumorigenesis is a complex process, during which inherited factors are interconnected with numerous environmental risks in the predisposition to this disease.^1^ In addition to a plethora of genetic components that control DNA repair, cell proliferation and apoptosis,^2^ several acquired risks for oral cancer, including viral infection^3^ and habitual consumption of tobacco, alcohol and areca nuts,^4^, ^5^, ^6^ have been recognised. Moreover, other causes of oral cancer may involve but not limited to poor oral hygiene, periodontitis,^7^ rehearsal micro traumatismes caused by sharp edges of decayed teeth and chronic infections and inflammation.^8^ These etiological parameters unveil that oral cancer is of a multifactorial nature that, to some extent, affects or is altered by a unique microbial milieu in the oral cavity. This notion has been currently supported by extensive investigations of shifts in the makeup and function of oral microbiota,^9^ implicating a causal relationship between oral microbiome and tumorigenesis via direct metabolism of carcinogens and induction of inflammation.^10^ Oral squamous cell carcinoma (OSCC) is the most common type of oral cancer^11^, ^12^, ^13^ and associated with high mortality^14^ despite the availability of current treatment options. Taking such heterogeneous nature of disease aetiology into consideration, all risk parameters mentioned above seem to be intertwined and required to assess the incidence and prognosis of OSCC.

Recent discoveries in the mechanisms and functions of long noncoding RNAs (lncRNAs) have led the field of functional genomics into a new era.^15^, ^16^, ^17^, ^18^ Until now, a considerable number of lncRNA genes are found to be linked to a myriad of pathological conditions,^19^ including malignant diseases. Among them, cyclin dependent kinase inhibitor 2B antisense RNA 1 (*CDKN2B‐AS1*), a lncRNA initially identified in a genome‐wide association study of type 2 diabetes and cardiovascular diseases,^20^ has been proposed as a promising therapeutic target or prognostic marker for various malignancies, as it is aberrantly expressed in diverse types of tumours.^21^ Functional investigations suggested that *CDKN2B‐AS1* promoted carcinogenesis by modulating the expression of tumour suppressor genes, *CDKN2A*/*CDKN2B*, in *cis*,^22^, ^23^ indicating a therapeutic potential for cancers. In addition to gene transcription, *CDKN2B‐AS1* controlled cancer development and treatment at post‐transcriptional levels by gene silencing via binding with a variety of microRNAs.^24^, ^25^, ^26^, ^27^ Intriguingly, *CDKN2B‐AS1* was shown to promote cancer cell survival and glucose metabolism in acute myeloid leukaemia through suppressing the expression of a key regulator of glucose uptake, adiponectin receptor 1.^28^ These observations collectively reveal that *CDKN2B‐AS1*, behaving as a scaffold, sponge or signal hub, could orchestrate cancer progression via genomic targeting, transcriptional regulation, epigenetic mechanisms and antisense interference.

Recently, studies using genome‐wide or targeted gene approaches have unveiled a genetic association of *CDKN2B‐AS1* single‐nucleotide polymorphism (SNP) with the predisposition to some cancer types. Specifically, *CDKN2B‐AS1* variants, rs10757274 and rs1333040, were linked to a lower likelihood of developing stage III–IV tumours in papillary thyroid cancer.^29^ *CDKN2B‐AS1* rs2151280 has been shown to affect susceptibility to basal cell carcinoma^30^ and progression‐free survival in multiple myeloma.^31^ Moreover, rs1412832 polymorphism demonstrated a genome‐wide significant association with increased risk of pancreatic cancer.^32^ *CDKN2B‐AS1* rs1011970 was reported as a risk variant for melanoma and correlated with reduced expression of *CDKN2B‐AS1*,^33^ whereas rs11515 polymorphism was connected to breast cancer and elevated expression of *CDKN2B‐AS1*.^34^ In addition, associations of rs4977756 and rs1412829 with glioma reached a genome‐wide threshold.^35^, ^36^, ^37^ Nevertheless, the interactive effect of *CDKN2B‐AS1* gene polymorphisms and habitual risk factors on oral cancer remains unclear. Here, we conducted a case–control study to evaluate the impact of *CDKN2B‐AS1* SNPs (rs564398, rs1333048, rs1537373, rs2151280 and rs8181047) on the risk of OSCC.

## 2 MATERIALS AND METHODS

### 2.1 Subjects

In this study, 1060 male patients with oral cancer and 1183 cancer‐free male controls were recruited to assess the impact of *CDKN2B‐AS1* gene polymorphisms on the risk of developing OSCC, with the approval by the institutional review board of Chung Shan Medical University Hospital in Taichung, Taiwan (CS1‐21151). Subjects, enrolled from 2012 to 2021, provided informed written consent at enrolment. Clinical staging and tumour differentiation of OSCC patients were evaluated by a pathologist and rated according to the TNM staging system of the American Joint Committee on Cancer (AJCC).^38^ Males who had neither self‐reported history of cancer of any site nor oral precancerous diseases, such as oral submucous fibrosis, leukoplakia, erythroplakia, verrucous hyperplasia, etc., were enrolled to the control cohort. Information regarding age and habitual risk factors (betel quid chewing, cigarette smoking and alcohol drinking) was obtained from each participant by using a questionnaire. Betel quid chewing and alcohol drinking are defined as behavioural use of areca nut‐related products and alcoholic beverages, respectively. Cigarette smoking is defined as current smoking of at least one cigarette per day during the latest 3 months.

### 2.2 *CDKN2B‐AS1* SNPs genotyping

Five SNPs from *CDKN2B‐AS1* gene were selected in this study according to their potential interactions with the predisposition to various disorders.^39^, ^40^, ^41^, ^42^, ^43^ DNA from the whole blood was isolated by using QIAamp DNA Blood Mini kit (Qiagen).^44^, ^45^ Evaluation of allelic discrimination for these five loci was carried out by using the TaqMan assay with an ABI StepOne™ Real‐Time PCR System (Applied Biosystems) and subsequently analysed by SDS version 3.0 software (Applied Biosystems).

### 2.3 Statistical analysis

Demographic and clinical parameters between OSCC cases and controls were compared by using Mann–Whitney *U* test. Relationship of genotype frequencies with the risk or clinical status of OSCC was explored by multiple logistic regression models followed by adjustment for potential confounding variables. The differences of CDKN2B‐AS1 levels in the head and neck squamous cell carcinoma (HNSCC) dataset from The Cancer Genome Atlas (TCGA) were compared by Mann–Whitney *U* test. Data were calculated by using SAS software (v9.4, 2013; SAS Institute Inc., Cary, NC). The threshold of difference or association was set by a *p* value of < 0.05.

## 3 RESULTS

### 3.1 Subject characteristics

The detail of OSCC characters are also presented in the Table 1. In this case–control study, 1060 male patients with OSCC and 1183 cancer‐free male controls were recruited to investigate the association of *CDKN2B‐AS1* gene polymorphisms with the development of oral tumorigenesis. Demographic and clinical features of both study cohorts were evaluated (Table 1). In compliance with the observations from other studies conducted in Central and Southeast Asia,^14^, ^46^ significant differences in the frequencies of cigarette smoking, alcohol consumption and betel quid chewing were detected between the case and control group. Among the case group, nodal spread and distal metastasis occurred in 33.3% and 0.7% of patients, respectively. A total of 84.8% of tumours were moderately or poorly differentiated in our study.

**TABLE 1:** Comparisons of clinical and demographic characteristics in patients with OSCC (*n* = 1060) and cancer‐free controls (*n* = 1183).

Variable	Controls (*N* = 1183)	Patients (*N* = 1060)	*p*‐Value
Age (yrs)			
≦55	603 (51.0%)	517 (48.8%)	0.299
>55	580 (49.0%)	543 (51.2%)	
Betel quid chewing			
No	988 (83.5%)	285 (26.9%)	
Yes	195 (16.5%)	775 (73.1%)	0.001*
Cigarette smoking			
No	554 (46.8%)	175 (16.5%)	
Yes	629 (53.2%)	885 (83.5%)	0.001*
Alcohol drinking			
No	948 (80.1%)	587 (55.4%)	
Yes	235 (19.9%)	473 (44.6%)	0.001*
Stage			
I + II		507 (47.8%)	
III + IV		553 (52.2%)	
Tumour T status			
T1 + T2		512 (48.3%)	
T3 + T4		548 (51.7%)	
Lymph node status			
N0		707 (66.7%)	
N1 + N2 + N3		353 (33.3%)	
Metastasis			
M0		1052 (99.3%)	
M1		8 (0.7%)	
Cell differentiation			
Well differentiated		161 (15.2%)	
Moderately or poorly differentiated		899 (84.8%)	

*Note*: Mann–Whitney *U* test was used between controls and patients with oral cancer.

* *p*‐Value < 0.05 as statistically significant.

### 3.2 Association of CDKN2B‐AS1 SNP with the progression of oral cancer

To test the possible association of *CDKN2B‐AS1* gene polymorphisms with the development of OSCC, five SNPs of *CDKN2B‐AS1* gene (rs564398, rs1333048, rs1537373, rs2151280 and rs8181047) were genotyped in this study. The distribution of genotype and allele frequencies for each SNP in OSCC patients and cancer‐free controls was examined. No deviation (*p* > 0.05) from Hardy–Weinberg equilibrium in the controls was detected for all SNPs tested. We failed to observe any significant association of these SNPs with the risk of oral cancer between the case and control group (Table 2). Furthermore, we evaluated the correlations of polymorphic genotypes of *CDKN2B‐AS1* with clinicopathological characteristics of OSCC patients. We observed that patients who carry at least one minor allele (C) of rs1333048 (CA and CC) were more prone to develop late‐stage (stage III/IV, AOR, 1.480; 95% CI, 1.129–1.940; *p* = 0.005) and large‐size (greater than 2 cm in the greatest dimension, AOR, 1.347; 95% CI, 1.028–1.765; *p* = 0.031) tumours as compared with those homologous for the major allele (AA) and (Table 3). These results reveal a potential link of *CDKN2B‐AS1* gene polymorphisms with the disease progression but not the occurrence in oral cancer.

**TABLE 2:** Association of *CDKN2B‐AS1* genotypes/alleles with the risk of OSCC.

Variable	Controls (*N* = 1183)	Patients (*N* = 1160)	AOR (95% CI)^a^
rs564398			
TT	938 (79.3%)	847 (79.9%)	1.000 (reference)
TC	232 (19.6%)	192 (18.1%)	1.029 (0.792–1.337)
CC	13 (1.1%)	21 (2.0%)	1.801 (0.764–4.248)
TC + CC	245 (20.7%)	213 (20.1%)	1.071 (0.832–1.381)
T allele	2108 (89.1%)	1886 (89.0%)	1.000 (reference)
C allele	258 (10.9%)	234 (11.0%)	1.106 (0.878–1.393)
rs1333048			
AA	324 (27.4%)	296 (27.9%)	1.000 (reference)
AC	612 (51.7%)	516 (48.7%)	0.788 (0.618–1.006)
CC	247 (20.9%)	248 (23.4%)	1.061 (0.794–1.418)
AC + CC	859 (72.6%)	764 (72.1%)	0.865 (0.689–1.087)
A allele	1260 (53.3%)	1108 (52.3%)	1.000 (reference)
C allele	1106 (46.7%)	1012 (47.7%)	1.016 (0.880–1.174)
rs1537373			
GG	474 (40.1%)	434 (40.9%)	1.000 (reference)
GT	569 (48.1%)	481 (45.4%)	0.981 (0.787–1.222)
TT	140 (11.8%)	145 (13.7%)	1.307 (0.943–1.811)
GT + TT	709 (59.9%)	626 (59.1%)	1.044 (0.848–1.285)
G allele	1517 (64.1%)	1349 (63.6%)	1.000 (reference)
T allele	849 (35.9%)	771 (36.4%)	1.094 (0.941–1.271)
rs2151280			
AA	514 (43.5%)	496 (46.8%)	1.000 (reference)
AG	526 (44.5%)	446 (42.1%)	0.896 (0.721–1.113)
GG	143 (12.0%)	118 (11.1%)	0.858 (0.612–1.202)
AG + GG	669 (56.5%)	564 (53.2%)	0.888 (0.723–1.090)
A allele	1554 (65.7%)	1438 (67.8%)	1.000 (reference)
G allele	812 (34.3%)	682 (32.2%)	0.914 (0.784–1.066)
rs8181047			
GG	898 (75.9%)	804 (75.9%)	1.000 (reference)
GA	261 (22.1%)	233 (22.0%)	1.113 (0.869–1.425)
AA	24 (2.0%)	23 (2.1%)	0.966 (0.466–1.999)
GA + AA	285 (24.1%)	256 (24.1%)	1.100 (0.866–1.397)
G allele	2057 (86.9%)	1841 (86.8%)	1.000 (reference)
A allele	309 (13.1%)	279 (13.2%)	1.074 (0.867–1.331)

^a^ The adjusted odds ratio (AOR) with their 95% confidence intervals were estimated by multiple logistic regression models after controlling for betel quid chewing, cigarette smoking and alcohol drinking.

**TABLE 3:** Clinical statuses and *CDKN2B‐AS1* rs1333048 genotype frequencies in OSCC patients.

Variable	AA (*N* = 296)	AC + CC (*N* = 764)	AOR (95% CI)	*p*‐Value
Clinical stage				
Stage I + II	162 (54.7%)	345 (45.2%)	1.00 (reference)	0.005*
Stage III + IV	134 (45.3%)	419 (54.8%)	1.480 (1.129–1.940)	
Tumour size				
≦T2	158 (53.4%)	354 (46.3%)	1.00 (reference)	0.031*
>T2	138 (46.6%)	410 (53.7%)	1.347 (1.028–1.765)	
Lymph node metastasis				
No	205 (69.3%)	502 (65.7%)	1.00 (reference)	0.255
Yes	91 (30.7%)	262 (34.3%)	1.184 (0.886–1.582)	
Metastasis				
M0	295 (99.7%)	757 (99.1%)	1.00 (reference)	0.347
M1	1 (0.3%)	7 (0.9%)	2.746 (0.335–22.506)	
Cell differentiation				
Well	50 (16.9%)	111 (14.5%)	1.00 (reference)	0.289
Moderate or poor	246 (83.1%)	653 (85.5%)	1.219 (0.845–1.758)	

*Note*: Cell differentiation grade: grade I, well differentiated; grade II, moderately differentiated; grade III, poorly differentiated. The adjusted odds ratio (AOR) with their 95% confidence intervals were estimated by multiple logistic regression models after controlling for betel quid chewing, cigarette smoking and alcohol drinking.

* *p*‐Value < 0.05 as statistically significant.

### 3.3 Joint effect of CDKN2B‐AS1 rs1333048 and habitual risk factors on OSCC progression

As a genetic association of *CDKN2B‐AS1* rs1333048 with OSCC progression was noted, we further tested whether there is any combined effect of rs1333048 and three major habitual risks (cigarette smoking, betel quid chewing and alcohol drinking) on clinicopathological features of OSCC. For patients who chewed betel quid (*n* = 775) or smoked (*n* = 885), a significant association of rs1333048 genotypes (CA + CC) with the development of late‐stage tumours (stage III/IV) was detected in comparison with homozygotes for the major allele (AA) and (AOR, 1.480; 95% CI, 1.076–2.035; *p* = 0.016, for betel quid chewers; Table 4) and (AOR, 1.531; 95% CI, 1.136–2.063; *p* = 0.005, for cigarette smokers; Table 5). However, this association was not seen in patients who were not exposed to betel quid chewing (Table 4) or cigarette smoking (Table 5). Our data indicate that habitual exposure to behavioural risks in combination with *CDKN2B‐AS1* polymorphisms might influence OSCC progression.

**TABLE 4:** Clinical statuses and genotypic frequencies of *CDKN2B‐AS1* rs1333048 in OSCC patients who are betel quid chewers or not betel quid chewers.

Variable	Betel quid chewers (*N* = 775)				Non‐betel quid chewers (*N* = 285)			
	AA	AC + CC	AOR (95% CI)	*p*‐Value	AA	AC + CC	AOR (95% CI)	*p*‐Value
	(*N* = 211)	(*N* = 564)			(*N* = 85)	(*N* = 200)		
Clinical stage								
Stage I + II	117 (55.5%)	258 (45.7%)	1.00 (reference)	0.016*	45 (52.9%)	87 (43.5%)	1.00 (reference)	0.136
Stage III + IV	94 (44.5%)	306 (54.3%)	1.480 (1.076–2.035)		40 (47.1%)	113 (56.5%)	1.482 (0.884–2.483)	
Tumour size								
≦T2	113 (53.6%)	270 (47.9%)	1.00 (reference)	0.164	45 (52.9%)	84 (42.0%)	1.00 (reference)	0.065
>T2	98 (46.4%)	294 (52.1%)	1.254 (0.912–1.723)		40 (47.1%)	116 (58.0%)	1.628 (0.970–2.733)	
Lymph node metastasis								
No	148 (70.1%)	375 (66.5%)	1.00 (reference)	0.324	57 (67.1%)	127 (63.5%)	1.00 (reference)	0.583
Yes	63 (29.9%)	189 (33.5%)	1.189 (0.843–1.677)		28 (32.9%)	73 (36.5%)	1.163 (0.678–1.997)	
Metastasis								
M0	211 (100.0%)	559 (99.1%)	1.00 (reference)	–	84 (98.8%)	198 (99.0%)	1.00 (reference)	0.859
M1	0 (0.0%)	5 (0.9%)	2.746 (0.335–22.506)		1 (1.2%)	2 (1.0%)	0.801 (0.070–9.203)	
Cell differentiation								
Well	38 (18.0%)	92 (16.3%)	1.00 (reference)	0.575	12 (14.1%)	19 (9.5%)	1.00 (reference)	0.250
Moderate or poor	173 (82.0%)	472 (83.7%)	1.127 (0.743–1.709)		73 (85.9%)	181 (90.5%)	1.578 (0.726–3.432)	

*Note*: Cell differentiation grade: grade I, well differentiated; grade II, moderately differentiated; grade III, poorly differentiated. The adjusted odds ratio (AOR) with their 95% confidence intervals were estimated by multiple logistic regression models after controlling for cigarette smoking and alcohol drinking.

* *p*‐Value < 0.05 as statistically significant.

**TABLE 5:** Clinical statuses and genotypic frequencies of *CDKN2B‐AS1* rs1333048 in OSCC patients who are cigarette smokers or not cigarette smokers.

Variable	Cigarette smokers (*N* = 885)				Non‐cigarette smokers (*N* = 175)			
	AA	AC + CC	AOR (95% CI)	*p*‐Value	AA	AC + CC	AOR (95% CI)	*p*‐Value
	(*N* = 241)	(*N* = 644)			(*N* = 55)	(*N* = 120)		
Clinical stage								
Stage I + II	134 (55.6%)	290 (45.0%)	1.00 (reference)	0.005*	28 (50.9%)	55 (45.8%)	1.00 (reference)	0.448
Stage III + IV	107 (44.4%)	354 (55.0%)	1.531 (1.136–2.063)		27 (49.1%)	65 (54.2%)	1.289 (0.669–2.481)	
Tumour size								
≦T2	132 (54.8%)	312 (48.5%)	1.00 (reference)	0.095	26 (47.3%)	42 (35.0%)	1.00 (reference)	0.112
>T2	109 (45.2%)	332 (51.5%)	1.288 (0.957–1.734)		29 (52.7%)	78 (65.0%)	1.699 (0.883–3.267)	
Lymph node metastasis								
No	166 (68.9%)	424 (65.8%)	1.00 (reference)	0.388	39 (70.9%)	78 (65.0%)	1.00 (reference)	0.390
Yes	75 (31.1%)	220 (34.2%)	1.150 (0.837–1.582)		16 (29.1%)	42 (35.0%)	1.363 (0.673–2.762)	
Metastasis								
M0	240 (99.6%)	638 (99.1%)	1.00 (reference)	0.450	55 (100.0%)	119 (99.2%)	1.00 (reference)	–
M1	1 (0.4%)	6 (0.9%)	2.266 (0.271–18.962)		0 (0.0%)	1 (0.8%)	0.801 (0.070–9.203)	
Cell differentiation								
Well	42 (17.4%)	101 (15.7%)	1.00 (reference)	0.531	8 (14.6%)	10 (8.3%)	1.00 (reference)	0.216
Moderate or poor	199 (82.6%)	543 (84.3%)	1.135 (0.764–1.686)		47 (85.4%)	110 (91.7%)	1.875 (0.693–5.070)	

*Note*: Cell differentiation grade: grade I, well differentiated; grade II, moderately differentiated; grade III, poorly differentiated. The adjusted odds ratio (AOR) with their 95% confidence intervals were estimated by multiple logistic regression models after controlling for betel quid chewing and alcohol drinking.

* *p*‐Value < 0.05 as statistically significant.

### 3.4 Clinical insights of CDKN2B‐AS1 in head and neck cancer

**FIGURE 1:**
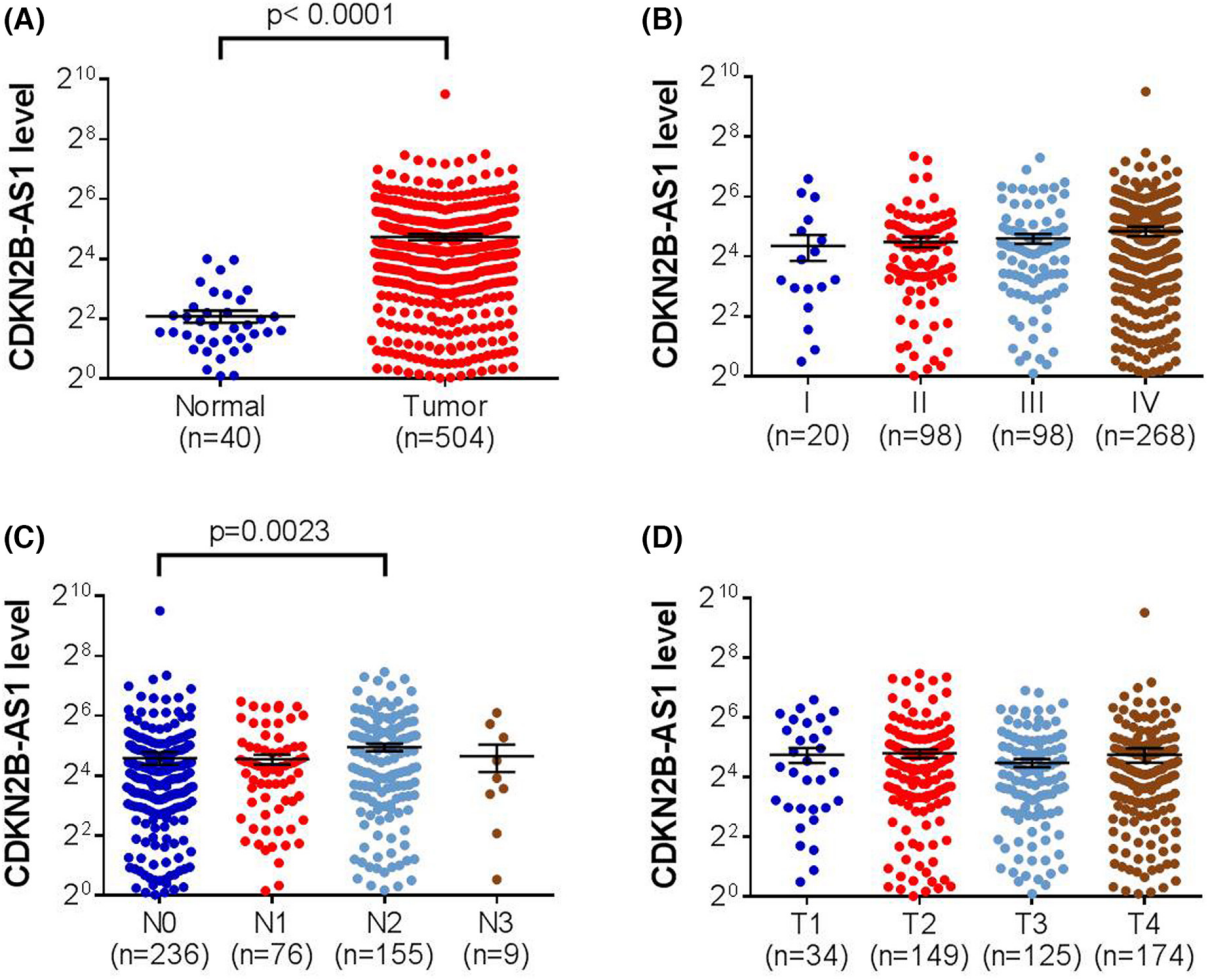
CDKN2B‐AS1 expression levels are increased and associated with clinicopathological parameters in head and neck squamous cell carcinoma (HNSCC). (A) Comparison of CDKN2B‐AS1 expression between tumour and normal tissues. (B–D) Correlations of CDKN2B‐AS1 expression with the clinical staging (B), lymphatic spread (C) and tumour size (D) of HNSCC from The Cancer Genome Atlas (TCGA) database. The total number of samples is given in brackets. *p*‐Values are calculated with student's *t*‐test.

Since a genetic impact of CDKN2B‐AS1 on OSCC was seen, its clinical relevance was explored by conducting extra surveys using public datasets. We showed an increase of CDKN2B‐AS1 expression in patients with head and neck squamous cell carcinoma (HNSCC) in The Cancer Genome Atlas (TCGA) dataset (Figure 1). Furthermore, elevated CDKN2B‐AS1 levels were associated with lymph node metastasis in HNSCC patients. These results reflect a link of CDKN2B‐AS1 induction to HNSCC progression.

## 4 DISCUSSION

It is generally accepted that the development and progression of oral cancer are affected by a combination of etiological factors, both inherited and acquired. Here, an association of *CDKN2B‐AS1* variants, rs1333048, with the development of late‐stage tumours but not with the disease occurrence was demonstrated in oral cancer. Further stratification analyses revealed that this correlation of rs1333048 genotypes with the advanced stage of disease was only seen in habitual betel quid or cigarette users but not in patients free of these major habitual risks. Our results unveil an interactive effect of *CDKN2B‐AS1* rs1333048 with habitual exposure to behavioural risks on OSCC progression.

Like several SNPs of *CDKN2B‐AS1*, rs1333048 was extensively documented for its correlation with many types of malignant diseases, such as breast,^47^ thyroid^29^ and prostate cancer.^48^ However, instead of conferring the susceptibility to oral malignancies, we observed a joint effect of rs1333048 with habitual risk factors on OSCC progression to the advanced stage of disease. Intriguingly, a genetic association of rs1333048 with periodontitis has been reported^49^ and successfully replicated in additional cohorts,^50^, ^51^ indicating a potential link of *CDKN2B‐AS1* variants to oral health. Periodontitis is an inflammatory condition of the oral mucosa and epidemiologically associated with some chronic inflammation‐driven comorbidities, including oral cancer.^52^ In addition, rs1333048 has been found to be associated with the levels of highly sensitive C‐reactive protein (hsCRP), which is a biomarker for systemic inflammation.^53^ These evidence together with our findings led us to tentatively postulate a mechanistic linkage between the aberration of tissue‐specific CDKN2B‐AS1 functions and overlapping disease phenotypes of chronic inflammation.^54^ Although the functional analysis of rs1333048 remains unavailable, several diseased associated *CDKN2B‐AS1* SNPs were shown to affect its free energy of folding, activity of enhancer regions, capacity of miRNA sponge and access to chromatins, leading to predicted alterations in secondary structure, abundance or downstream functionality.^55^ Collectively, our findings highlight an interplay between *CDKN2B‐AS1* variants and habitual risks in the promotion of tissue‐specific inflammatory responses, potentiating the progression of oral cancer.

Current studies of expression quantitative trait locus (eQTLs) have demonstrated that most lncRNA gene expression is influenced by genetic variations.^56^ Through surveying the HNSCC data from TCGA, we noted a link of *CDKN2B‐AS1* induction with the cancer risk and lymphatic spread. Although it is unclear whether rs1333048 serves as an eQTL, specific genotypes of other *CDKN2B‐AS1* variants were found to manipulate the expression of *CDKN2B‐AS1* gene. For instance, rs10811656, rs1333049, rs2383208 and rs10811661 were found to increase or decrease *CDKN2B‐AS1* expression.^33^, ^57^ These loci were located within a predicted enhancer region in the downstream of the *CDKN2B‐AS1* gene and distal to its exon 21. Due to the proximity of rs1333048 to these alleles located within a predicted or proven enhancer, it implies that *CDKN2B‐AS1* rs1333048 may alter its own expression to affect OSCC progression. In addition, another group of *CDKN2B‐AS1* variants only impact *CDKN2A/B* expression but not *CDKN2B‐AS1* expression.^58^, ^59^ *CDKN2A/B* are well‐known tumour suppressor genes that have been implicated in many hallmarks of oral cancer^60^ and frequently altered in OSCC genome.^61^, ^62^ Taken together, these SNPs may function as eQTLs in a cell or tissue type‐specific manner to influence oral tumorigenesis.

Moreover, some disease associated *CDKN2B‐AS1* SNPs were reported to interfere with its RNA splicing and subsequently affect the stability of RNA isoforms.^63^ In lymphocytes, rs10757278 A allele was found to impede the skipping of exon 15, prompting the formation of a circular transcript that is repellent to RNase‐mediated cleavage. This circular form of *CDKN2B‐AS1* RNA can elicit nucleolar stress and p53 activation, leading to the induction of apoptosis and inhibition of proliferation.^64^ These findings reveal another potential function of *CDKN2B‐AS1* SNPs in modulating oral cancer progression.

Our study demonstrated a joint effect of *CDKN2B‐AS1* gene polymorphisms and habitual risk factors on the development of late‐stage OSCC; nevertheless, extra investigations are needed to address several limitations in the study. One is that quantitative definition for major habitual risks (betel nut chewing, alcohol drinking and cigarette smoking) was unavailable, which might underestimate the impacts of these environmental risks on the development and progression of oral cancer. Another issue is that the molecular mechanism underlying the role of rs1333048 in OSCC progression remains a puzzle, as the functionality of many other *CDKN2B‐AS1* SNPs has been documented in other tissue types.^31^, ^65^, ^66^, ^67^ Whether the variation (A > C) affects its own expression or disrupts the interaction with PRC or microRNAs needs further explorations. Moreover, our findings here may be restricted to particular ethnic populations if not replicated in other cohorts.

In conclusion, our results exhibit an association of *CDKN2B‐AS1* rs1333048 with the development of late‐stage tumours but not with the disease occurrence in oral cancer. This correlation of rs1333048 genotypes with the advanced stage of disease was only observed in habitual betel quid or cigarette users but not in patients without exposure to these major habitual risks. These findings suggest an interactive effect of *CDKN2B‐AS1* rs1333048 with habitual consumption of betel quid‐related products or cigarettes on OSCC progression.

## AUTHOR CONTRIBUTIONS

**Jung‐Chun Yeh:** Conceptualization (equal); writing – original draft (equal); writing – review and editing (equal). **Yi‐Tzu Chen:** Conceptualization (equal); writing – original draft (equal); writing – review and editing (equal). **Ying‐Erh Chou:** Methodology (equal). **Shih‐Chi Su:** Methodology (equal). **Lun‐Ching Chang:** Methodology (equal). **Yen‐Lin Chen:** Writing – original draft (equal). **Chiao‐Wen Lin:** Conceptualization (equal); writing – original draft (equal); writing – review and editing (equal). **Shun‐Fa Yang:** Conceptualization (equal); writing – original draft (equal); writing – review and editing (equal).

## FUNDING INFORMATION

This research was funded by Chung Shan Medical University Hospital, Taiwan (CSH‐2022‐C‐019).

## CONFLICT OF INTEREST STATEMENT

The authors declare that there is no conflict of interest.

## Data Availability

The data used to support the findings of the present study are available from the corresponding author upon request.
